# Molecular Characterization of Fecal Extended-Spectrum β-Lactamase- and AmpC β-Lactamase-Producing *Escherichia coli* From Healthy Companion Animals and Cohabiting Humans in South Korea

**DOI:** 10.3389/fmicb.2020.00674

**Published:** 2020-04-15

**Authors:** Jun Sung Hong, Wonkeun Song, Hee-Myung Park, Jae-Young Oh, Jong-Chan Chae, Seri Jeong, Seok Hoon Jeong

**Affiliations:** ^1^Department of Laboratory Medicine, Research Institute of Bacterial Resistance, Yonsei University College of Medicine, Seoul, South Korea; ^2^Department of Laboratory Medicine, College of Medicine, Hallym University, Chuncheon, South Korea; ^3^Department of Veterinary Internal Medicine, College of Veterinary Medicine, Konkuk University, Seoul, South Korea; ^4^Division of Biotechnology, College of Environmental and Bioresource Sciences, Chonbuk National University, Iksan, South Korea

**Keywords:** *Escherichia coli*, CTX-M, CMY-2, healthy companion animal, human, copy number

## Abstract

This study aimed to describe the distribution and characterization of fecal extended-spectrum β-lactamase (ESBL)- and AmpC-producing *Escherichia coli* isolates from healthy companion animals and cohabiting humans. A total of 968 rectal swab samples from 340 participants, including healthy companion animals and cohabiting humans, were collected from 130 households in South Korea from 2018 to 2019. To determine the bacterial profiles of the participants, several experiments were performed as follows: antimicrobial susceptibility testing, PCR and direct sequencing for ESBL/AmpC production, PFGE, MLST, whole genome sequencing and qRT-PCR. A total of 24.9 and 21.5% of the *E. coli* isolates from healthy companion animals and cohabiting humans were ESBL/AmpC producers, respectively. The *bla*_CTX–M–__14_ gene was the most prevalent ESC resistance gene in both pets (*n* = 25/95, 26.3%) and humans (*n* = 44/126, 34.9%). The *bla*_CMY–__2_ gene was also largely detected in pets (*n* = 19, 20.0%). Overall, intrahousehold pet-human sharing of ESBL/AmpC *E. coli* isolates occurred in 4.8% of households, and the isolates were all CTX-M-14 producers. In particular, ten CMY-2-producing *E. coli* isolates from seven dogs and three humans in the different households belonged to the same pulsotype. The MIC values of cefoxitin and the transcription level in CMY-2-producing *E. coli* isolates were proportional to the *bla*_CMY–__2_ copy number on the chromosome. Our results showed that the clonal spread of fecal ESBL/AmpC-producing *E. coli* households’ isolates between healthy companion animals and cohabiting humans was rare, but it could happen. In particular, *E. coli* ST405 isolates carrying multiple *bla*_CMY–__2_ genes on the chromosome was sporadically spread between companion animals and humans in South Korea.

## Introduction

The close contact between companion animals and humans may cause bacterial transmission across animals or humans by horizontal transfer (indirect transmission) and clonal spread (direct transmission) (Damborg et al., 2016). The majority of studies have focused on the transmission of human-to-companion animal with hospitalized or infected (Bogaerts et al., 2015), but few studies have focused on the fecal transmission of bacteria between healthy companion animals and cohabiting humans as categorized households (Marques et al., 2019).

Since the late 1900s, third- and fourth-generation extended-spectrum cephalosporin (ESC)-resistant *Enterobacteriaceae* have been frequently reported in both human and veterinary fields (Schmiedel et al., 2014; Lee et al., 2018). ESC resistance in *Enterobacteriaceae* is often associated with extended-spectrum β-lactamase (ESBL) and AmpC β-lactamase production, mainly CTX-M-type for ESBL in *Enterobacteriaceae*, CMY-type for AmpC in *Escherichia coli*, and DHA-type for AmpC in *Klebsiella pneumoniae* (Lartigue et al., 2004; Verdet et al., 2009; Kameyama et al., 2013). In addition, a wide diversity of carbapenemase-producing *Enterobacteriaceae* has been also found worldwide (Gauthier et al., 2018).

CMY-2 is the most prevalent AmpC β-lactamase in ESC-resistant *E. coli* isolates in animals and humans worldwide (Ewers et al., 2012; Lazarus et al., 2015). The dissemination of the *bla*_CMY–__2_ gene largely corresponds to self-transferable IncA/C or IncI1 plasmids (Ben Sallem et al., 2014; Guo et al., 2014), but chromosomal *bla*_CMY–__2_ gene transfer through specific IS*Ecp1*-mediated transposition is also responsible for the spread of the *bla*_CMY–__2_ gene (Verdet et al., 2009; Mata et al., 2012). The transcription of the *bla*_CMY–__2_ gene could be altered by a putative promoter within IS*Ecp1*, the correlation between IS*Ecp1* and CMY-2 called the spacer sequence, and the copy number of the *bla*_CMY–__2_ gene (Smet et al., 2008; Ma et al., 2011; Kurpiel and Hanson, 2012; Manageiro et al., 2015).

Our previous study targeted “diseased” companion animals (Hong et al., 2019), while this study focused on “healthy” companion animals. Namely, the significant difference between our two studies is the study population. This study aimed to compare the distribution and molecular relatedness of fecal ESBL/AmpC-producing *E. coli* isolates collected from healthy companion animals and cohabiting humans.

## Materials and Methods

### Study Population

The study population included households with healthy companion animals and humans living together. All human participants were informed of the main goals of this study and were asked to sign a consent form before inclusion. Participants who were considered “healthy” were included if they had no bacterial infection or did not take antibiotics in the previous month. A participant household was made up of healthy companion animals (a dog or a cat) and one or two cohabiting humans (a veterinarian hospital staff and/or a staff’s family). A total of 340 participants (101 dogs, 38 cats, 130 vet’s staff, and 71 staff’s families) living in 130 households belonging to 18 veterinarian hospitals were enrolled for 2018–2019 (Supplementary Material). All human participants were more than 16 years old, and 70% were women. The age of companion animals ranged from less than 1 to 16 years, and 43% were female (data not shown).

### Specimen Collection and Bacterial Isolates

This study was carried out in accordance with the recommendations of the ethnic guidelines of KonKuk University College of Veterinary Medicine, South Korea. Individual written informed consent for the use of samples was obtained from all the vet’s staff and the staff’s families.

Enrolled humans collected their own rectal swab samples and rectal swab samples from coliving companion animals into sterile containers. The sample collection was performed with one, two, three, four, and/or five repetitions (first in January 2018, second in March 2018, third in September 2018, fourth in February 2019, and/or fifth in April 2019) per participant (Supplementary Material). Specimens were refrigerated until processing. The rectal swab samples plated onto CHROMagar ESBL plates (CHROMagar, Paris, France) were incubated at 36°C for 18 h. Up to three suspected *E. coli* colonies (dark pink to reddish colonies) were isolated, and a single colony was subcultured onto a blood agar plate. The subcultured colonies were confirmed as *E. coli* by matrix-assisted laser desorption ionization-time of flight mass spectrometry (MALDI-TOF MS) with a Vitek-MS (bioMerieux, Marcy-Etoile, France).

### Antimicrobial Susceptibility Testing

Antimicrobial susceptibility to ampicillin, piperacillin, ampicillin-sulbactam, cefazolin, cefoxitin, cefotaxime, ceftazidime, cefepime, aztreonam, ertapenem, imipenem, meropenem, amikacin, gentamicin, ciprofloxacin, and trimethoprim-sulfamethoxazole was tested by the agar disk diffusion method on cation-adjusted Mueller-Hinton agar (Difco Laboratories, Detroit, MI, United States). For further characterization, the minimum inhibitory concentrations (MICs) of cefoxitin, cefotaxime, and ceftazidime were determined by the broth microdilution method according to the CLSI guidelines (CLSI, 2019), and the MICs of colistin were assessed by the broth microdilution method using Mueller–Hinton broth (Difco Laboratories), following the criteria of EUCAST (2019). *E. coli* ATCC 25922 and *Pseudomonas aeruginosa* ATCC 27853 were used for quality control.

### Identification of Antimicrobial Resistance Determinants and the *bla*_CMY–__2_ Promoter

Template DNA was prepared by the boiling method. *bla*_CTX–M–__1__–like_, *bla*_CTX–M–__2__–like_, *bla*_CTX–M–__9__–like_, and *bla*_CTX–M–__25__–like_ for CTX-M-type ESBLs were amplified by PCR and direct sequencing in cefotaxime-non-susceptible *E. coli* isolates; *bla*_DHA_, *bla*_ACC_, *bla*_ACT_, and *bla*_FOX_ for AmpCs were amplified by PCR and direct sequencing in cefoxitin-non-susceptible *E. coli* isolates; IMP-, VIM-, NDM-, KPC-, GES-, and OXA-48-like for carbapenemases were amplified by PCR and direct sequencing in carbapenem-non-susceptible *E. coli* isolates: 1138 bp region upstream of the *bla*_CMY–__2_ gene within IS*Ecp1* and the 116 bp spacer sequence as the predicted *bla*_CMY–__2_ promoter between IS*Ecp1* and CMY-2 for *bla*_CMY–__2_ promoter as described previously (Smet et al., 2008; Ma et al., 2011; Kurpiel and Hanson, 2012; Manageiro et al., 2015; Hong et al., 2019). The sequences were compared to published DNA sequences using BLAST^1^.

### Epidemiological Bacterial Typing

The representative *E. coli* isolates carrying the five predominant ESBL/AmpC genes for epidemiological data analysis underwent pulsed-field gel electrophoresis (PFGE) with *Xba*I. *E. coli* ATCC 25922 was included as a quality control. Multilocus sequence typing (MLST) was performed on CMY-2-producing *E. coli* isolates belonged to the same pulsotype.

### Whole Genome Sequencing

The three CMY-2 producing *E. coli* ST405 isolates (E118, E597, and E686) among the same pulsotype were selected. Two (E597 and E686) of the three isolates were recovered from a dog and a human in this study, respectively. The other well-characterized isolate (E118) was recovered from a dog in our previous study in 2017 (Hong et al., 2019). The bacterial DNA was extracted from the three isolates using a Wizard Genomic DNA Purification kit (Promega, Madison, WI, United States). After extraction, DNA shearing was performed using a g-TUBE apparatus (Covaris, Inc., Moburn, MA, United States) and the fragments were purified by using 0.45× of the final volume of washed Agencourt AMPure XP magnetic beads (Beckman Coulter Inc., Brea, CA, United States) (Hong et al., 2016). For bacterial whole genome sequencing (WGS), single-molecule real-time sequencing was carried out on a PacBio RSII instrument (Pacific Biosciences, Menlo Park, CA, United States). The coding sequences, tRNA sequences, and rRNA sequences were annotated using the NCBI Prokaryotic Genome Annotation Pipeline^2^.

### Quantitative Reverse Transcription PCR

The expression level of the *bla*_CMY–__2_ gene in *E. coli* ST405 isolates belonged to the same pulsotype was evaluated by quantitative reverse transcription PCR (qRT-PCR) assays. The *E. coli* isolates were cultured in Muller–Hinton broth at 37°C with vigorous agitation until OD_600_ = 1.5–2, corresponding to the exponential phase. Bacterial RNA was extracted using the RNeasy Mini Kit (Qiagen, Hilden, Germany), and cDNA was synthesized using the TOPscript^TM^ cDNA Synthesis Kit (Enzynomics, Daejeon, South Korea). Amplification was carried out in a final volume of 20 μL containing 10 μL of premix (RbTaq^TM^ qPCR 2X preMIX; Enzynomics), 1 μL of each primer, 7 μL of distilled water, and 1 μL of cDNA with a CFX96 system (Bio-Rad, Hercules, CA, United States). Transcript measurement was carried out in quadruplicate, and relative quantification of the *bla*_CMY–__2_ gene was calculated with the 2^–Δ^ ^Δ^ ^CT^ method using 16S rRNA as a reference. The E118 strain was measured in every batch for comparison as a reference strain.

### Data Analysis

For PFGE, the unweighted-pair group method with averaging based on the Dice similarity coefficient and a clustering algorithm was used to create a dendrogram. Isolates with >80% PFGE similarity were considered as shared clones (Tenover et al., 1995). Statistical analysis was performed using SPSS Statistics version 24.0.0 (IBM Corp., Armonk, NY, United States). The chi-square test was used for comparisons between groups, with a *p* value of less than 0.05 considered statistically significant.

## Results

### ESBL/AmpC Genotypes and Antimicrobial Susceptibility

A total of 968 rectal swab samples from healthy companion animals (291 dogs and 91 cats) and their cohabiting humans (381 vet’s staff and 205 of staff’s families) were collected from 340 participants (Supplementary Table S1).

A total of 221 ESBL/AmpC-producing *E. coli* isolates, including 95 (24.9%, 95/382) healthy companion animals and 126 (21.5%, 126/586) cohabiting humans in the 130 households, were isolated. CTX-M-14 (*n* = 25, 26.3%) was the most common ESBL/AmpC β-lactamase in companion animals. CMY-2 (*n* = 19, 20.0%) was the second most predominant ESBL/AmpC β-lactamase, followed by CTX-M-55 (*n* = 12, 12.6%), CTX-M-15 (*n* = 9, 9.5%), and CTX-M-27 (*n* = 7, 7.4%). The *bla*_CTX–M–__14_ gene (*n* = 44, 34.9%) was also the most common ESC-resistance gene in cohabiting humans, followed by *bla*_CTX–M–__15_ (*n* = 24, 19.0%), *bla*_CTX–M–__27_ (*n* = 21, 16.7%), *bla*_CTX–M–__55_ (*n* = 8, 6.3%), and *bla*_CMY–__2_ (*n* = 8, 6.3%). CTX-M-1 and CTX-M-24 were found only in companion animals, while CTX-M-3, CTX-M-65, and CMY-48 were detected only in humans. Several *E. coli* isolates cocarrying ESBL/AmpC genes (producing ESBL + ESBL, ESBL + AmpC, or AmpC + AmpC) were also observed (Table 1). Carbapenemases (IMP-, VIM-, NDM-, KPC-, GES-, and OXA-48-like) were not detected in two imipenem-intermediate *E. coli* isolates (H7h3) along with CTX-M-15 and DHA-1 recovered from vet’s staff and staff’s family.

**TABLE 1:** Genotypes of ESBL- and AmpC-producing *E. coli* isolates from healthy companion animals and cohabiting humans.

**ESBL**	**AmpC**	**No. (%) of isolates**					
		**Companion animals**			**Cohabiting humans**		
		**Dog**	**Cat**	**Total**	**Vet’s staff**	**Staff’s family**	**Total**
		**(*n* = 291)**	**(*n* = 91)**	**(*n* = 382)**	**(*n* = 381)**	**(*n* = 205)**	**(*n* = 586)**
CTX-M-1		2	0	2	0	0	0
CTX-M-3		0	0	0	1	1	2
CTX-M-3 + CTX-M-14		3	0	3	0	0	0
CTX-M-15		8	1	9	19	6	25
CTX-M-15 + CTX-M-14		0	0	0	2	1	3
CTX-M-15 + CTX-M-27		0	0	0	1	0	1
CTX-M-15	CMY-2	2	1	3	0	0	0
CTX-M-15	DHA-1	0	0	0	1	1	2
CTX-M-55		11	0	11	8	0	8
CTX-M-55 + CTX-M-27		0	0	0	1	2	3
CTX-M-55 + CTX-M-65		1	0	1	0	0	0
CTX-M-55	CMY-2	1	0	1	1	0	1
CTX-M-24		3	0	3	0	0	0
CTX-M-27		4	3	7	18	3	21
CTX-M-27	CMY-2	0	1	1	0	0	0
CTX-M-64		2	3	5	1	0	1
CTX-M-65		0	0	0	1	1	2
CTX-M-65	CMY-2	0	0	0	1	0	1
CTX-M-65	DHA-1	0	0	0	1	0	1
CTX-M-14		19	6	25	30	14	44
CTX-M-14	CMY-2	2	0	2	0	0	0
CTX-M-14	DHA-1	0	0	0	0	1	1
	CMY-2	19	0	19	4	2	6
	CMY-2 + DHA-1	2	0	2	0	0	0
	CMY-48	0	0	0	1	0	1
	DHA-1	1	0	1	3	0	3
Total		80 (27.5)	15 (16.5)	95 (24.9)	94 (24.7)	32 (15.6)	126 (21.5)

**FIGURE 1:**
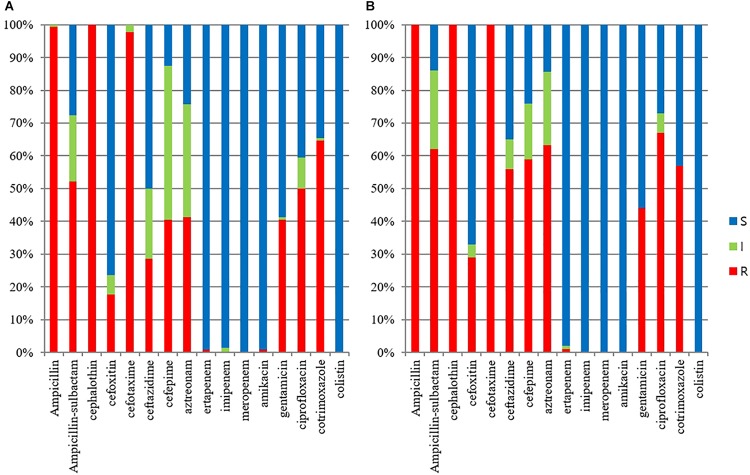
Antimicrobial susceptibilities of ESBL/AmpC-producing *E. coli* veterinary isolates. **(A)** *E. coli* isolates from humans (*n* = 125); **(B)** *E. coli* isolates from companion animals (*n* = 95).

The ESBL/AmpC-producing *E. coli* isolates in pets showed a statistically lower susceptibility rate to ampicillin-sulbactam, cefoxitin, ceftazidime, cefepime, aztreonam, gentamicin, and ciprofloxacin than those in cohabiting humans but showed a statistically higher susceptibility rate to trimethoprim-sulfamethoxazole than those in cohabiting humans (*p* value <0.05). All isolates in companion animals and cohabiting humans were susceptible to colistin (Figure 1).

### Epidemiological Relatedness Categorized in Households

A total of 130 non-duplicated *E. coli* isolates carrying the five predominant ESBL/AmpC genes (49 of CTX-M-14, 25 of CTX-M-15, 15 of CTX-M-27, 16 of CTX-M-55, and 25 of CMY-2) in the 84 households were selected for subjecting to PFGE analysis.

**FIGURE 2:**
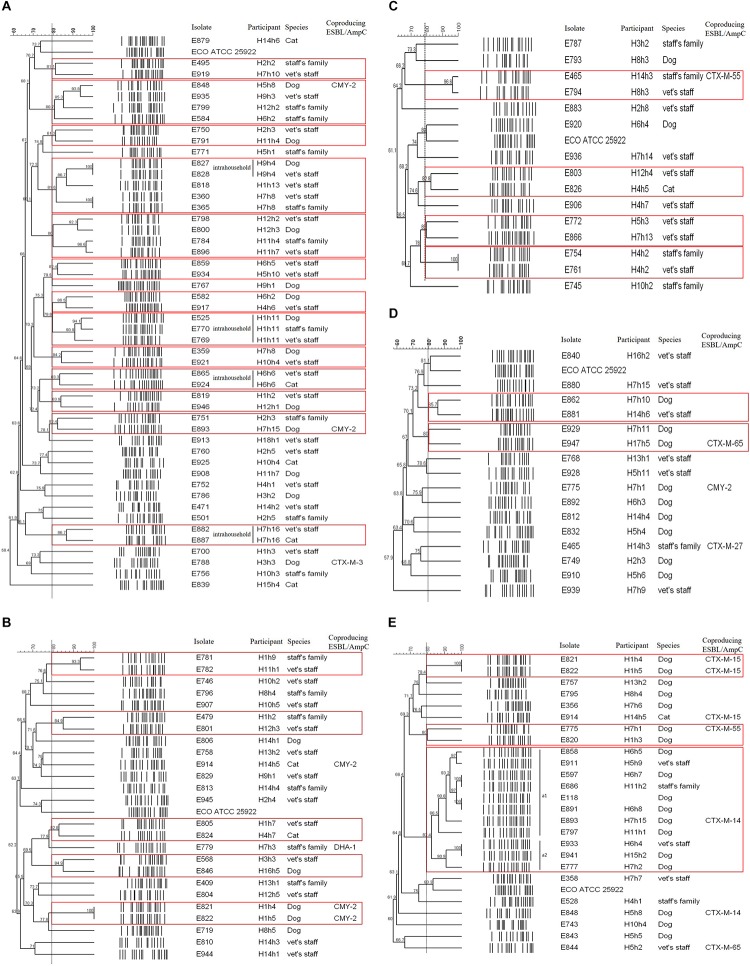
PFGE dendrogram of ESBL/AmpC-producing *E. coli* isolates by genotype. **(A)** CTX-M-14; **(B)** CTX-M-15; **(C)** CTX-M-27; **(D)** CTX-M-55; **(E)** CMY-2. The red box indicates the same pulsotype (>80% similarity) as the closely related case.

Of the 49 *E. coli* isolates carrying the *bla*_CTX–M–__14_ gene, 13 cases were considered as shared clones belonged to the same pulsotype. Four cases in four households occurred as intrahousehold pet-human sharing (H9h4, H1h11, H6h6, and H7h16), indicating belonged to same household and same veterinary hospital with >80% clonal similarity. Seven cases occurred as interhousehold pet-human sharing (Figure 2A).

Of the 25 *E. coli* isolates carrying the *bla*_CTX–M–__15_ gene, five cases had closed bacterial strain typing (cladogram sharing >82%), and intrahousehold pet-human sharing was absent. Two cases of them were observed: interhousehold dog-human sharing and interhousehold dog-dog sharing (Figure 2B).

Of the 15 CTX-M-27-producing *E. coli* isolates, four cases showed shared clones without intrahousehold pet-human sharing. One case of them was observed as interhousehold cat-human sharing (Figure 2C).

Of the 16 *E. coli* isolates carrying the *bla*_CTX–M–__55_ gene, two cases were considered as shared clones without intrahousehold pet-human sharing, but each case occurred as interhousehold dog-human sharing and interhousehold dog-dog sharing (Figure 2D).

Of the 25 *E. coli* isolates carrying the *bla*_CMY–__2_ gene, three cases showed closed bacterial strain typing (cladogram sharing >80%) without intrahousehold pet-human sharing. Two cases of them were occurred as interhousehold dog-dog sharing. In the remaining one case, eleven *E. coli* isolates (eight dogs and three humans from different households) corresponded to ST405 by MLST analysis were included in one cluster (a) by PFGE (pulsotype a1 (six dogs and two humans) with >86% clonal similarity and pulsotype a2 (two dogs and one human) with >90% clonal similarity), similar to the well-characterized E118 strain (*E. coli* ST405 canine isolate carrying the *bla*_CMY–__2_ gene) in our previous report (Hong et al., 2019; Figure 2E).

Overall, intrahousehold pet-human sharing was 4.8% (four of the 84 households), and these shared isolates were all CTX-M-14 producers.

### Genetic Profiles of CMY-2-Producing *E. coli* ST405 Isolates

The bacterial profile was further characterized, and 11 CMY-2-producing *E. coli* ST405 isolates belonged to the a1 and a2 pulsotypes were divided into three groups based on their MIC values to cefoxitin: 256 mg/L was included in group 1, 128 mg/L was included in group 2, and 64 mg/L was included in group 3 (Table 2).

**TABLE 2:** MIC and *bla*_CMY–__2_ expression levels in the 11 CMY-2-producing *E. coli* ST405 isolates.

**Isolate**	**Household**	**Species**	**Coproducing ESBL/AmpC**	**MIC**			**Relative CMY-2 transcript level ± SD compared to E118 strain**
				**FOX**	**CTX**	**CAZ**	
E686	H11h2	Human	No	64	16	32	0.223 ± 0.003
E911	H5h9	Human	No	64	32	64	0.188 ± 0.007
E933	H6h4	Human	No	64	32	64	0.307 ± 0.024
E118	(3)*	Dog	No	128	32	64	1
E797	H11h1	Dog	No	128	32	64	1.385 ± 0.059
E891	H6h8	Dog	No	128	64	256	1.594 ± 0.079
E893	H7h15	Dog	CTX-M-14	128	256	64	1.935 ± 0.139
E597	H6h7	Dog	No	256	32	64	5.389 ± 0.238
E777	H7h2	Dog	No	256	64	128	7.374 ± 0.312
E858	H6h5	Dog	No	256	32	64	4.221 ± 0.530
E941	H15h2	Dog	No	256	32	64	5.324 ± 0.362

Abbreviations: Ct, cycle threshold; ST, sequence type; FOX, cefoxitin; CTX, cefotaxime; CAZ, ceftazidime; SD, standard deviation. An asterisk (*) indicates Reference 3 in this study.

**FIGURE 3:**
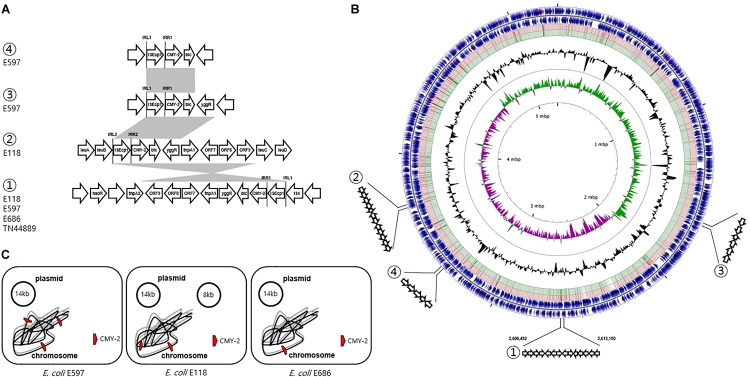
Genetic overview of three *E. coli* ST405 isolates carrying chromosomally located *bla*_CMY–2_. **(A)** Comparison of the genetic regions surrounding the *bla*_CMY–2_ gene. The shaded regions indicate the identical sequences sharing whatever same or converse orientation. **(B)** Circular genome map representation. The outermost ring: E118 open-reading frames on both forward and reverse strands. Red and green circle represents the blast results of E597 and E686 with E118 representing the positions covered by the BLASTN alignment, respectively. G + C content, black peak; G + C positive skew, green peak; G + C negative skew, purple peak. ① Region of genetic environmental structure from 2,600,492-2,613,180 bp in the E118, E597, and E686 strains identical to *E. coli* TN44889; ② region from 3,350,844-3,353,575 bp in E118 strain; ③ region from 1,669,367-1,672,220 bp in E597, and ④ region from 3,350,844-3,353,575 bp in E597 strain. **(C)** The chromosome and plasmid of three CMY-2-producing *E. coli* isolates.

Three isolates (E118, E597, and E686) were selected from each of the three groups, and WGS was carried out. The E597 strain (dog origin) in group 1 was composed of a 5.2 Mb circularized chromosome carrying three copies of *bla*_CMY–__2_ and two plasmids. The first plasmid of the RepFIB type had 176 CDSs, including only the *TEM-1* gene in ca. 14.4 kb. The second repA-type plasmid had 93 CDSs without the antimicrobial resistance (AMR) gene in ca. 8.3 kb. The E118 strain (dog origin) in group 2 was composed of a 5.3 Mb circularized chromosome carrying two copies of *bla*_CMY–__2_ and one plasmid. The RepFIB replicon plasmid had 175 CDSs, including *TEM-1* and *tet* for tetracycline resistance in ca. 14 kb. The E686 strain (human origin) in group 3 was composed of a 5.2 Mb circularized chromosome carrying one *bla*_CMY–__2_ and one plasmid. The plasmid of the RepFIB type had 191 CDSs, which included *TEM-1*, *tet*, and *ant1*, for streptomycin resistance in ca. 14.4 kb (Figure 3).

The genetic environment of the first *bla*_CMY–__2_ gene by IS*Ecp1*-mediated, truncated by IS*4*, in the E118 strain was *bla*_CMY–__2_-*blc-yggR*-*tnp1*-*orf7*-*orf8*-*orf9*-*tnp2*-*hsdR*. One inverted repeat was located IS*Ecp1* left (IRL1, GGATCTAAGATGCA), and the paired inverted repeat was IS*Ecp1* right (IRR1, TGCACCTTAAATCC). The second *bla*_CMY–__2_ gene sequence by IS*Ecp1*-mediated, adjacent to IRL2 (CCTAGATTCTACGT) and IRR2 (ACGTGGAATTTAGG), was conversely comprised in a different genetic organization (*bla*_CMY–__2_-*blc-yggR*-*tnp1*-*orf7*-*orf8*). This context was devoid of the last three CDSs (*orf9*-*tnp2*-*hsdR*) compared to the first *bla*_CMY–__2_-containing region. These three genes were identical to a part of the *Shigella flexneri* serotype 2a SRL pathogenicity island, being first reported to be involved in the transfer of antibiotic resistance (Luck et al., 2001). The paired 13 bp sequences for IRL1 and IRR1 adjacent to the configuration (IS*Ecp1*-*bla*_CMY–__2_) in the E597 strain were identical in the following three flanking regions in different locations on the chromosome: (i) *bla*_CMY–__2_-*blc*-*yggR*, (ii) *bla*_CMY–__2_-*blc-yggR*-*tnp1*-*orf7*-*orf8*-*orf9*-*tnp2*-*hsdR*, and (iii) *bla*_CMY–__2_-*blc*. In the E686 strain, the genetic context containing-the *bla*_CMY–__2_ gene by IS*Ecp1*-mediated, adjacent to IRL1 and IRR1, was identified as *bla*_CMY–__2_-*blc-yggR*-*tnp1*-*orf7*-*orf8*-*orf9*-*tnp2*-*hsdR*, which was identical to the genetic context of the first *bla*_CMY–__2_ gene region in the E118 strain and the second *bla*_CMY–__2_ gene region in the E597 strain. The common chromosomal genetic regions (*bla*_CMY–__2_-*blc-yggR*-*tnp1*-*orf7*-*orf8*-*orf9*-*tnp2*-*hsdR*) in the E118, E597, and E686 strains were inserted in the same location, which was from 2,600,492 to 2,613,180 bp (Figure 3).

### Evaluation of *bla*_CMY–__2_ Transcript Levels

The gene expression of *bla*_CMY–__2_ in group 1 was substantially elevated from 4.221- to 7.374-fold compared to the E118 strain. Group 2 and group 3 exhibited 1.385- to 1.935-fold and 0.188- to 0.307-fold increases in *bla*_CMY–__2_ expression, respectively, compared to the E118 strain (Table 2).

### *bla*_CMY–__2_ Promoter Evaluation

**FIGURE 4:**
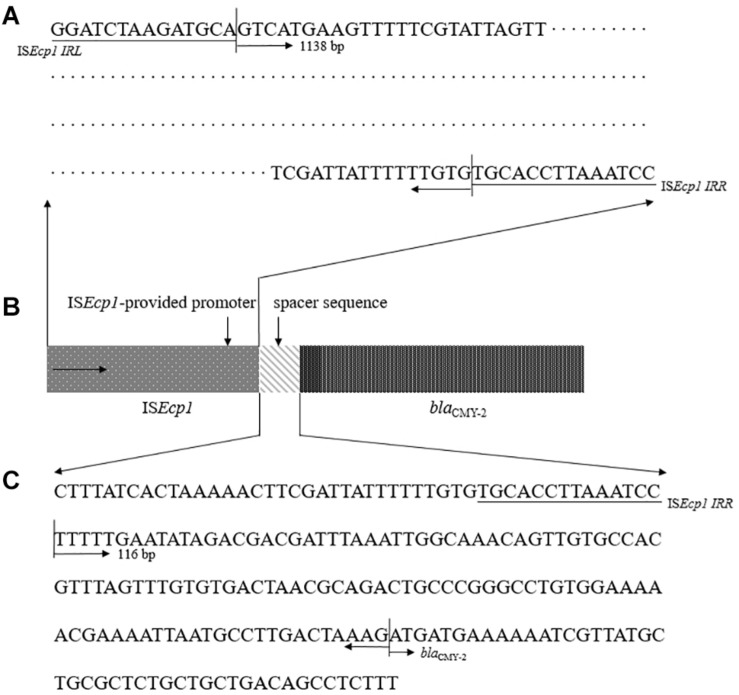
Representative configuration of *bla*_CMY–2_ promoters. **(A)** IS*Ecp1*-provided putative promoter region sequence. **(B)** Structure of the *bla*_CMY–2_ containing region by IS*Ecp1*-mediated transposition. **(C)** sequence analysis as predicted promoter within the 116 bp spacer between IS*Ecp1* and *bla*_CMY–2_.

We evaluated two sequences previously presumed to be involved in high levels of ESBL/AmpC gene expression (Smet et al., 2008; Ma et al., 2011; Kurpiel and Hanson, 2012; Manageiro et al., 2015): IS*Ecp1*-provided promoter region sequence and a spacer sequence in front of *bla*_CMY–__2_ (Figure 4). For the examined sequences, all 11 CMY-2-producing *E. coli* ST405 isolates shared 100% identity for above two putative promoter sequences related to high levels of ESBL/AmpC gene expression.

## Discussion

In our previous study, CMY-2 was the most prevalent ESBL/AmpC enzyme in diseased companion animals, accounting for 26.4% of the ESBL/AmpC enzymes, and CTX-M-14 and CTX-M-55 were the second most abundant enzymes. In addition, NDM-5 was detected in four *E. coli* isolates from four dogs along with *bla*_CTX–M–__15_ and *bla*_CMY–__2_ (Hong et al., 2019). However, in this study, CTX-M-14 was equally highest in healthy companion animals and cohabiting humans, and no carbapenemase genes were detected. Interestingly, the rate of ESBL/AmpC-producing *E. coli* isolates recovered from healthy companion animals and cohabiting humans was high, observing in more than 20% of the total rectal swab samples. The fact that companion animals could play an important role as a reservoir of AMR bacteria to humans has been reported previously (Day, 2011). In this regard, colonized feces of healthy companion animals can be considered a carrier for potentially transferring ESBL and/or AmpC β-lactamase for ESC resistance to humans.

To analyze clonal relatedness among predominant ESBL/AmpC-producing *E. coli* isolates from healthy companion animals and cohabiting humans, several closely related clonal lineages belonged to the same pulsotype were observed. Of them, intrahousehold pet-human sharing of AMR bacteria was observed in four cases (4.3%) of the 130 households. Therefore, this result showed that AMR households’ isolate could be shared between companion animals and humans, which was considered intrahousehold pet-human sharing, while largely occurred by interhousehold pet-human sharing with unknown routes of transmission of *E. coli* isolates colonization.

The clonal relationship of eleven CMY-2-producing *E. coli* ST405 isolates belonged to the a1 and a2 pulsotypes showed cladograms sharing >82%, but the isolates belonged to different households. In other words, these cases were considered interhousehold pet-human sharing. Chromosomal concordance between three CMY-2-producing *E. coli* ST405 isolates (E118, E597, and E686) was high (>98%), and prophage sequences were also identical by WGS analysis. Although they shared one identical genetic environment (*bla*_CMY–__2_-*blc-yggR*-*tnp1*-*orf7*-*orf8*-*orf9*-*tnp2*-*hsdR*), which was identical to the genetic context of the clinical isolate *E. coli* TN44889 in 2004 (Verdet et al., 2009), the E118, E597, and E686 strains had different *bla*_CMY–__2_ copy numbers and different *bla*_CMY–__2_-containing regions. In this regard, our findings shed doubt whether the three *E. coli* ST405 isolates were considered as clonal spread based on PFGE analysis, or sporadic spread based on WGS analysis. This is important because if WGS methodology is used, the clonal relatedness of the strains could be different compared to methodology based on PFGE. This doubt demonstrates that epidemiological questions regarding the mechanism for dissemination of resistance determinants should be clarified to better understand the epidemiology of bacteria (de Been et al., 2014). In this study, intrahousehold pet-human sharing cases are likely to be considered as cases by clonal spread, while interhousehold cases seem to be sporadic spread.

We initially prospected the *bla*_CMY–__2_ gene as the often observed plasmid-mediated AmpC β-lactamase (Verdet et al., 2009; Kameyama et al., 2013), but the locations of three *bla*_CMY–__2_ genes on the chromosome were confirmed by WGS analysis. In the remaining *E. coli* ST405 isolates, the *bla*_CMY–__2_ gene was suspected to be a chromosomal gene. This is because there are two identical 13 bp sequences (paired IRL1 and IRR1) adjacent to IS*Ecp1* and similar antimicrobial susceptibility phenotypes in 11 CMY-2-producing *E. coli* ST405 isolates. This result suggested that integration of the *bla*_CMY–__2_-containing environment into the chromosome of certain ST405 clone appears to be involved in the dissemination of cefoxitin-resistant *E. coli* in South Korea.

CTX-M-type ESBL, especially CTX-M-15, is commonly more dominant than CMY-type AmpC β-lactamases in humans (Lee et al., 2018), but CMY-2-producing *E. coli* isolates have been frequently detected as much as CTX-M-type in veterinary medicine worldwide (Pietsch et al., 2018; Donà et al., 2019; Hong et al., 2019). Interestingly, cefoxitin is often used in the veterinary field for treating gram-positive and anaerobic bacterial infections (Brook et al., 2013; Lappin et al., 2017). It is likely that veterinary gram-negative isolates can also become resistant by multiple transpositions of the *bla*_CMY–__2_ gene owing to cefoxitin selective pressure and then may accelerate the dissemination of cefoxitin-resistant *E. coli*.

For the three classified groups in 11 CMY-2-producing *E. coli* ST405 isolates, regarding the relationship between the MIC values of cefoxitin and the transcription level, the MIC values of cefoxitin were proportional to the *bla*_CMY–__2_ copy number, varying from one to three copies. However, no mutations were observed in the 1138 bp upstream sequence of the *bla*_CMY–__2_ gene and the 116 bp spacer sequence (Smet et al., 2008; Ma et al., 2011; Kurpiel and Hanson, 2012; Manageiro et al., 2015). This finding indicated that the higher level of *bla*_CMY–__2_ expression correlated with multiple copy numbers of the *bla*_CMY–__2_ gene is a key factor rather than the *bla*_CMY–__2_ promoter mutation, but it could not be excluded a limitation that the total number of CMY-2-producing *E. coli* ST405 isolates analyzed in this study was only eleven.

Unfortunately, we initially tried to isolate samples up to five times per participants during the collection procedure to enhance the accuracy of the study, but the number of sampling actually varied from one to five due to individual circumstances of the participants.

### Conclusion

This study presented the intrahousehold pet-human sharing of fecal ESBL/AmpC-producing *E. coli* isolates in healthy companion animals and cohabiting humans, indicating that the colonized feces of healthy companion animals could be a reservoir for spreading AMR bacteria to humans. Notably, *E. coli* ST405 isolates carrying multiple *bla*_CMY–__2_ genes on the chromosome sporadically spread between companion animals and humans in South Korea. In addition, the gene copy number on the chromosome seems to be the most important factor affecting the transcription level of the AMR gene.

## Data Availability Statement

The datasets generated for this study can be found in the NCBI GenBank: https://www.ncbi.nlm.nih.gov/nuccore/cp049196; https://www.ncbi.nlm.nih.gov/nuccore/cp049197; https://www.ncbi.nlm.nih.gov/nuccore/cp049198.

## Ethics Statement

This study was carried out in accordance with the recommendation of ethical guidelines of KonKuk University College of Veterinary Medicine, South Korea. Written informed consent was obtained from the owners for the participation of their animals in this study.

## Author Contributions

WS is the corresponding author who was in charge of the study design, data analysis and proofreading of the manuscript. JH performed an examination of molecular work (resistance gene, PFGE, and MLST), analyzed the experimental data, and wrote the manuscript. H-MP, J-CC, and SHJ designed the sample collection and the experiments. J-YO designed the experiments and performed the experiment. SJ conducted the statistical analysis.

## Conflict of Interest

The authors declare that the research was conducted in the absence of any commercial or financial relationships that could be construed as a potential conflict of interest.

## Footnotes

1 https://blast.ncbi.nlm.nih.gov/Blast.cgi

2 http://www.ncbi.nlm.nih.gov/books/NBK174280
